# Effect of combined oral contraceptive use on verbal memory function in healthy women

**DOI:** 10.1007/s00737-025-01592-z

**Published:** 2025-05-19

**Authors:** Mette C. Hochheim, Vibe G. Frokjaer, Søren V. Larsen, Vibeke H. Dam

**Affiliations:** 1 Neurobiology Research Unit, Copenhagen University Hospital Rigshospitalet, Copenhagen, Denmark; 2 Department of Clinical Medicine, Faculty of Health and Medicinal Sciences, University of Copenhagen, Copenhagen, Denmark; 3 Psychiatric Center Copenhagen, Mental Health Services in the Capital Region of Denmark Copenhagen, Copenhagen, Denmark

**Keywords:** Combined oral contraceptives, Cognition, Verbal affective memory, Reproductive mental health, Sex steroid hormones

## Abstract

**Purpose:**

Female sex hormones as well as the synthetic hormones contained within combined oral contraceptives (COCs) may influence emotional and cognitive functioning including learning and memory; however, findings are inconsistent. We here present the largest study to date investigating the effect of COC use on verbal memory in healthy women.

**Methods:**

COC use and verbal memory scores were available from the CIMBI database for 205 healthy women in the reproductive age. We assessed if verbal memory and affective bias differed between COC users and non-users. In a subgroup of natural cycling women in the follicular phase, we assessed if verbal memory was associated with plasma estradiol levels.

**Results:**

We found no statistically significant group differences in either overall memory performance (*p* = 0.16) or affective memory bias (*p* = 0.18) between COC users and non-users, although there was a trend suggesting COC users may exhibit slightly better recall for short-term (*p* = 0.09) and long-term task (*p* = 0.08) conditions. Similarly, COC users tended to have slightly better overall memory compared with women in the follicular phase (*p* = 0.05). Follicular phase plasma estradiol levels were not associated with verbal memory.

**Conclusions:**

We found no support for COC use to negatively impact verbal memory, if anything COC users tended to perform better than natural cycling women in follicular phase; however, this could be influenced by a healthy user bias. In conclusion, these findings highlight that women who tolerate COCs well should not be concerned about potential adverse effects on memory.

**Supplementary Information:**

The online version contains supplementary material available at 10.1007/s00737-025-01592-z.

## Introduction

Approximately 151 million women use combined oral contraceptives (COCs) worldwide (United Nations 2019). While most research focuses on physical side effects to COCs, e.g. excessive risk of thromboembolic events, a significant group of women discontinue COC use due to mental health side effects (Martell et al. 2023). Therefore, there is growing recognition of the potential influence of COCs on mood and cognition (Gamsakhurdashvili et al. 2021; Gogos 2013; Lewis et al. 2019; Warren et al. 2014) including how they may affect brain structures and biology potentially involved in cognitive and emotional processes (Brønnick et al. 2020; Gogos et al. 2014; Larsen et al. 2020; Spalek et al. 2019; Warren et al. 2014). Specifically verbal memory appears to be affected by COC use with reports of enhanced verbal memory performance in COC user relative to non-users (Gogos et al. 2014; Gurvich et al. 2023; Warren et al. 2014), though other studies have found no effect of COC use on verbal memory (Islam et al. 2008; Mordecai et al. 2008; Rosenberg and Park 2002).

The female brain is exposed to natural hormonal fluctuations of estradiol and progesterone throughout the menstrual cycle. Changes in sex hormone levels have been shown to affect cognition including memory (Sherwin 2012) with several studies reporting enhancement in verbal memory performance during the midluteal phase where natural levels of estradiol and progesterone are high (Gogos 2013; Hampson 1990; Maki et al. 2002; Sherwin 2012). In line with this, changes in estradiol levels have also been linked to structural changes in the amygdala, hippocampus and prefrontal cortex, brain regions supporting learning and memory functions as well as emotion processing (Gamsakhurdashvili et al. 2021; Pletzer et al. 2019a, b; Schuster & Jansen 2022).

COCs contain estrogen, mainly the synthetic ethinylestradiol, and a synthetic progesterone (Brynhildsen 2014). COC use disrupts follicular maturation and ovulation by suppressing the hypothalamic-pituitary–gonadal axis through the negative feedback actions of synthetic hormones, resulting in a downregulation of the endogenous ovarian sex hormone production (D’Arpe et al. 2016). Such changes of the hormonal environment may impact brain structure and function (Pletzer et al. 2019a, b), e.g. duration of COC use has been linked to increased hippocampal volume (Pletzer et al. 2019). Intriguingly, we recently showed an effect of COC use on serotonergic neurotransmission (Larsen et al. 2020), specifically the serotonin 4 receptor, which in turn has been implicated in verbal memory function in both healthy individuals and patients with depression (Dam et al. 2025; Köhler-Forsberg et al. 2023; Murphy et al. 2020). Together these findings suggest that COC use may impact verbal memory through multiple brain-related mechanisms.

Although verbal memory is one of the most consistent and robust predictors of psychosocial functioning (Buck et al. 2020), no large-scale study has yet been conducted to reliably determine the effect of COC use on verbal memory in healthy women. This study aimed to investigate the effect of hormonal contraceptive use on verbal memory in a large sample of 205 healthy women, particularly verbal memory performance, verbal affective memory bias and the possible linkage of plasma estradiol levels to verbal memory performance.

## Materials and methods

### Study population

Verbal memory scores and information about COC use was extracted for 205 healthy premenopausal women from the Center for Integrated Molecular Brain Imaging (Cimbi) database (Knudsen et al. 2016). Inclusion criteria were no history of psychiatric or significant somatic illness; no use of hormonal contraceptives other than COCs (e.g. estrogen patch, hormonal intrauterine device, mini pill); no sterilization/hysterectomy; no pregnancy; no use of morning-after-pill within one month before cognitive testing. Ninety women were COC users and 115 were non-users, i.e. naturally cycling women in both the follicular and luteal phase. Neuropsychological data was acquired from studies conducted at the Neurobiological Research Unit at Copenhagen University Hospital, Rigshospitalet, Denmark, from 2011–2022 in accordance with the Declaration of Helsinki. The Ethics Committee in the capital of Denmark approved all study protocols ((KF)01–2006-20, (KF)23,830, (KF)01280377, H-1–2010-085, H-15004506, H-15017713, H-16026898, H-18038325, H-2–2010-108, H-2–2014-070, H-3–2012-083, H4-2011–103, H-4–2012-105) and all participants provided written informed consent. The study was preregistered at https://aspredicted.org/ (registration ID #144,949) before data was obtained from the Cimbi database.

### Hormonal contraceptive use and menstrual cycle phase

Information about hormonal contraceptive use was obtained by written questionnaire about COCs specifically and/or face-to-face interview about general contraceptive use within 30 days from the cognitive test day. Forty-two COC users had provided information about which COC type was used (see supplementary Figure S1) whereas the remaining 48 women reported using COCs but did not specify which type. In addition, information about menstrual cycle phase was obtained from 79 non-users, of whom 71 were in the follicular phase. Cycle phase was determined using the backward-count method based on the expected day of next menstruation calculated from the average menstrual cycle length and first day of last menstruation (Schmalenberger et al. 2021). Blood sample measurements of plasma estradiol levels acquired 0–1 days from the cognitive testing were available for 56 of the 71 women in the follicular phase. Two plasma estradiol samples were excluded due to plasma progesterone levels above 5 ng/mL, suggesting that the women were in their luteal phase (Leiva et al. 2015). Additionally, seven plasma estradiol samples were excluded due to non-compatible analysis methods. Thus, a total of 47 plasma estradiol samples were available from naturally cycling women in their follicular phase.

### Verbal memory

Verbal memory performance was assessed with the Verbal Affective Memory Task (VAMT) (Jensen et al. 2016). Data was pooled from the 24-word version (VAMT-24, *n* = 99) and the 26-word version (VAMT-26, *n* = 106) as we have previously shown that they can be used in a joint model (Hjordt et al. 2020). The VAMT is a word-list memory task that include positive, negative and neutral words and provides measures of immediate learning, short-term and long-term memory recall. For detailed information on instruction, administration and psychometric properties of VAMT-24/VAMT-26 see Hjordt et al. (Dam et al. 2021; Hjordt et al. 2020). The primary memory outcome was percentage correctly recalled words (Total Word Recall-%) across all valence categories (positive, negative and neutral) and conditions (immediate, short-term and long-term recall). Affective memory bias was used as a secondary outcome calculated as percentage correctly recalled negative words subtracted from percentage correctly recalled positive words across all conditions.

### Psychometrics

To assess subclinical mood symptoms, we used the Total Mood Disturbance (TMD) subscale from the self-report Profile of Mood State (POMS) questionnaire (Shahid et al. 2011). This scale includes 65 items rating negative mood states within the past week on Likert scale from 0 (“not at all”) to 4 (“very strong”). TMD scores range from −34 to 200 with higher scores reflecting a poorer overall mental state (Petrowski et al. 2021).

### Statistics

For our primary analysis, we used multiple linear regression to determine group differences between COC users (*n* = 90) and non-users (*n* = 115) in verbal memory performance (Total Word Recall-%). Planned post hoc analyses were also conducted for immediate, short-term and long-term recall memory components. As a sensitivity analyses, we excluded non-users whose hormonal contraceptive status had only been obtained based on self-report questionnaire data (*n* = 37) to mitigate any potential misclassification, as the self-report questionnaire only acquired information on COC use and not on other types of hormonal contraception.

For the secondary analyses, we used multiple linear regression models to assess a) whether subclinical mood symptoms and verbal memory performance differed between COC users (*n* = 90) and non-users (*n* = 114) by including TMD scores as a covariate in the primary analysis, b) if COC users (*n* = 90) and non-users (*n* = 115) differed in verbal affective bias, and c) if verbal memory performance was associated with plasma estradiol levels in naturally cycling women in the follicular phase (*n* = 47).

In a set of exploratory analyses, we estimated group differences in verbal memory performance between non-users (*n* = 115) and hormonal IUD users (*n* = 23) as well as between non-users in the follicular phase (*n* = 71) and COC users (*n* = 90). Lastly, we also tested for a potential age-dependent effect of COC use on verbal memory performance including interaction between age (categorized as 23 ≥ years vs > 23 years) and COC status (COC user vs non-user). All models were adjusted for age, educational level and VAMT-version.

## Results

### Participants

Demographic and psychometric data are presented in Table 1. Age ranged from 18 to 48 years, and non-users were averagely 1.72 years younger than COC users (*p* = 0.03). COC users had slightly lower total education score than non-users (*p* = 0.04), while no significant differences were found between groups for TMD-score (*p* = 0.94).

**Table 1:** Descriptive data

	COC users (*n* = 90)		Non-users (*n* = 115)		
	Mean (SD)	Range	Mean (SD)	Range	*p-value*
Age	26.50 (6.14)		24.78 (5.35)		0.03
Education	15.96 (1.59)		16.39 (1.28)		0.04
Subclinical mood symptoms (TMD)^a^	2.48 (16.19)		2.29 (16.70)		0.94
	n	%	n	%	
VAMT version (VAMT-26)	56	62.20%	34	37.80%	0.01

Group differences were assessed using Welch t-test for continuous variables and Chi-Square test for categorical variables. Subclinical mood was assessed with the Total Mood Disturbance (TMD). Education score was calculated by adding the participant’s total number of years in school to their educational score measured on a 5-point Likert scale (0 = no vocational degree to 5 = graduate or professional degree). COC = Combined oral contraceptive; VAMT = Verbal affective memory task. ^a^Data was missing for one individual

### COC and verbal memory

**Fig. 1:**
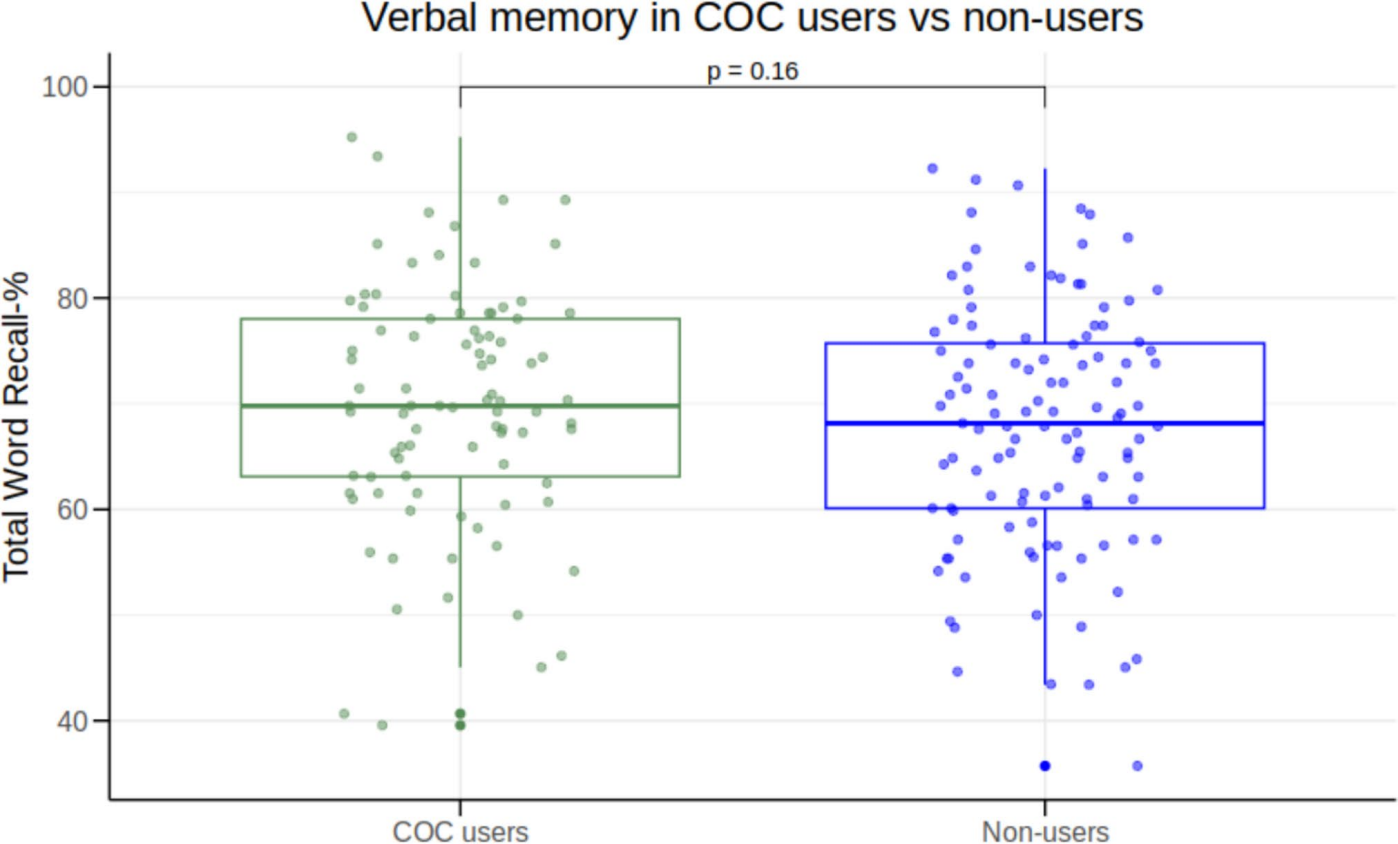
Group difference in verbal memory performance between combined oral contraceptive (COC) users (*n* = 90) and non-users (*n* = 115)

We found no significant difference in Total Word Recall-% (*β* = 2.30, *95% CI* [−0.94;5.54], *p* = 0.16) (Fig. 1), although COC users numerically outperformed non-users with 2.30 percentage point corresponding to ~ ½ word. We also found no statistically significant group difference in immediate (*β* = 1.70, *95% CI* [−1.33;4.73], *p* = 0.27), short-term (*β* = 3.87, *95% CI* [−0.56;8.29], *p* = 0.09) and long-term recall (*β* = 3.74, *95% CI* [−0.47;7.94], *p* = 0.08). In the sensitivity analysis constrained to non-users, whose contraceptive status was confirmed through face-to-face interview only, the overall estimated effect of COC use on verbal memory performance was similar (*β* = 2.33, *95% CI* [−1.29;5.94], *p* = 0.21).

### Subclinical mood symptoms and verbal memory

We found no evidence that TMD score was associated with Total Word Recall-% (*β* = 0.07, *95% CI* [−0.03;0.16], *p* = 0.18) nor did the inclusion of TMD scores change the overall estimated effect of COC use on verbal memory performance (see Table S1).

### COC and verbal affective memory bias

**Fig. 2:**
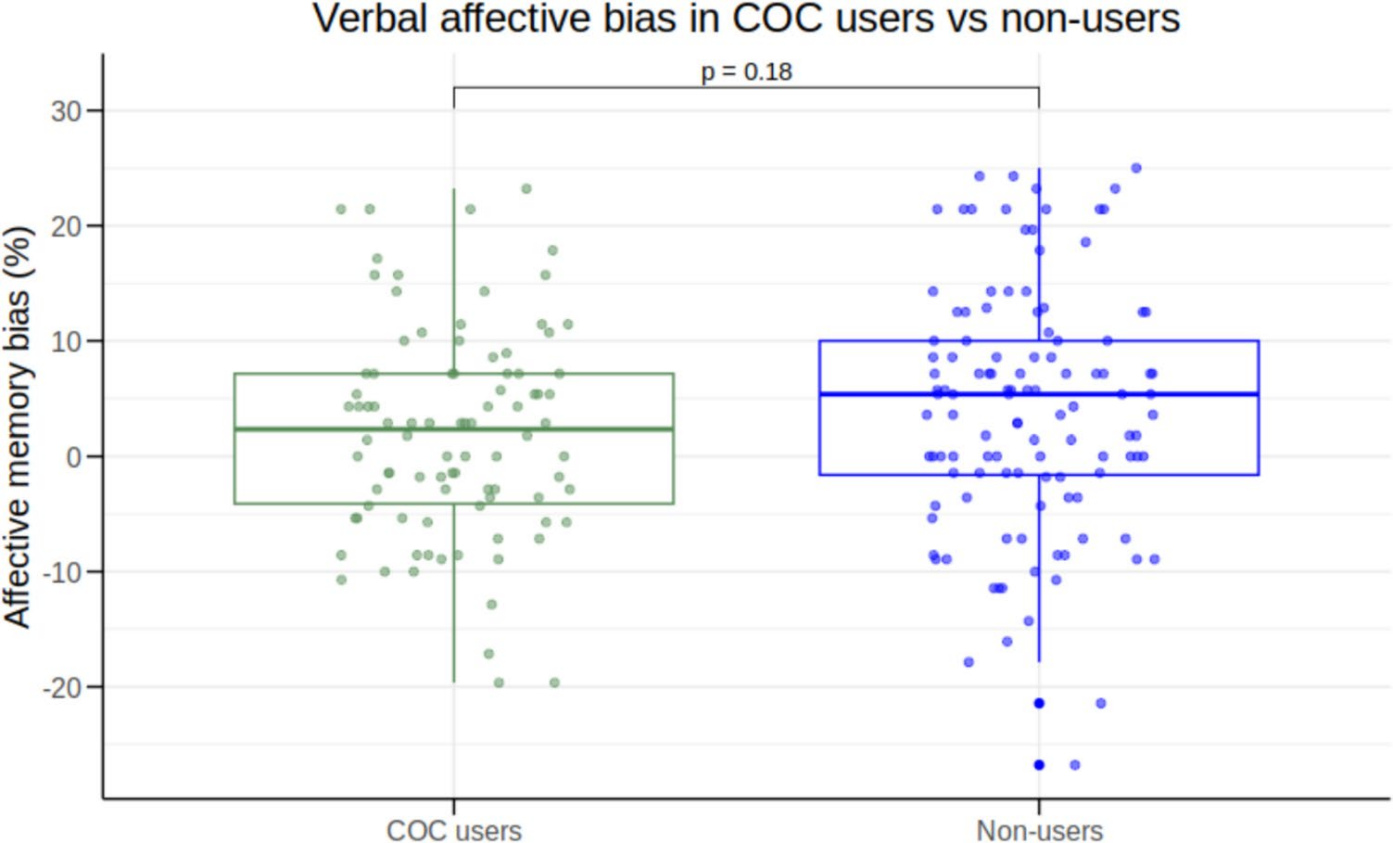
Group differences in verbal affective memory bias between combined oral contraceptive (COC) users (*n* = 90) and non-users (*n* = 115)

We found no evidence for a verbal affective memory bias in COC users relative to non-users (*β* = −1.97, *95% CI* [−4.86;0.92], *p* = 0.18) (Fig. 2). We also examined differences in immediate, short-term and long-term affective recall bias between the two groups and found no statistically significant differences (all *p* > 0.17).

### Plasma estradiol levels and verbal memory in the follicular phase

**Fig. 3:**
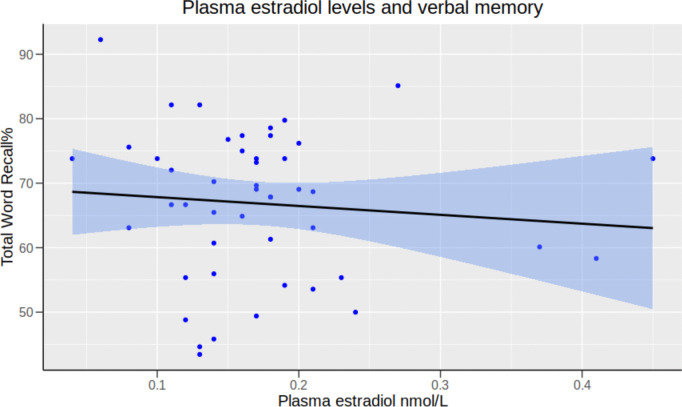
Association between plasma estradiol levels and Total Word Recall-% in naturally cycling women in the follicular phase of the menstrual cycle

Plasma estradiol levels were not statistically significantly associated with Total Word Recall-% in women in the follicular phase (*β* = −3.42, *95% CI* [−49.36;42.51], *p* = 0.88) (Fig. 3).

### Hormonal IUDs and verbal memory

We found no significant group difference in Total Word Recall-% in hormonal intrauterine device users relative to non-users (*β* = 2.32, *95% CI* [−3.17;7.81], *p* = 0.41).

### Verbal memory in the follicular phase

At a trend level, COC users performed slightly better in Total Word Recall-% than naturally cycling women in the follicular phase (*β* = 3.97, *95% CI* [−0.06;7.99], *p* = 0.053) corresponding to the recall of ~ 1 word more.

### Age-dependent effect of COC on verbal memory

We found no evidence for an age-dependent effect of COC use on verbal memory performance when participants were categorized by age (≤ 23 years vs > 23 years). The estimated effect of COC use on verbal memory performance were similar for both age groups ($\beta$= −0.47, *95% CI* [−6.14;7.08], *p* = 0.89). We found no evidence that one age group performed better than the other ($\beta$= −1.41, *95% CI* [−5.79;2.97], *p* = 0.53). Also, COC use had no significant effect on verbal memory performance in age group ≤ 23 years or age group > 23 years (both *p* > 0.28).

## Discussion

We here examine the putative effect of COC use on verbal memory in a large group of healthy women of reproductive age. We found no evidence of an overall difference between COC users and non-users in either verbal memory or verbal affective memory bias nor evidence of a mediating effect of the self-reported level of mental distress on verbal memory. Lastly, plasma estradiol levels were not significantly associated with verbal memory in naturally cycling women in the follicular phase.

The finding that COC users show comparable verbal memory performance as naturally cycling women is in line with previous studies (Islam et al. 2008; Mordecai et al. 2008; Rosenberg and Park 2002). However, while there was no statistically significant difference in overall verbal memory performance between COC users and non-users, post hoc analyses revealed a trend towards slightly better short-term and long-term memory capacity in COC users relative to non-users. It is therefore possible that COC use may impact retrieval of verbal memory some time after memory consolidation (Davignon et al. 2024; Gogos 2013). In support of this several studies have reported enhanced verbal memory in COC users compared to naturally cycling women both in word lists tasks (Gogos 2013; Mordecai et al. 2008) and more consistently in delayed recall of short stories (Gogos 2013; Jensen et al. 2023; Peragine et al. 2020). These pro-memory effect appears most prominent when COC users were compared to non-users in the follicular or periovulatory phase (Gogos 2013; Jensen et al. 2023; Peragine et al. 2020). Meanwhile, other studies found no evidence of an effect of COC use on verbal memory (Islam et al. 2008; Mordecai et al. 2008; Rosenberg and Park 2002) possibly due to small sample sizes or because the cognitive tasks used were designed to detect severe cognitive deficits in patient groups and may therefore not be sensitive enough to detect subtle changes in memory performance in healthy women. Together with our trend findings, this suggest that if present these putative pro-memory effects associated with COC use are small and likely have little impact on day-to-day functioning.

We found a tendency to a pro-memory effect of COC use when COC users were compared to naturally cycling women in the follicular phase, confirming what other studies have shown (Gogos 2013; Peragine et al. 2020). This indicates that the exposure to the synthetic steroids from COCs affects verbal memory performance differently compared to the influence from the endogenous hormone levels in the follicular phase, and thus indirectly that verbal memory performance may be influenced by the menstrual cycling phase as also supported by others’ finding of an enhanced verbal memory performance in the midluteal phase (Gogos 2013; Hampson 1990; Maki et al. 2002, Sherwin 2012). However, we did not observe any association between verbal memory and plasma estradiol levels in naturally cycling women in the follicular phase. We therefore speculate that verbal memory may primarily be influenced by progestogens either alone or in combination with an estrogen component. In support of this, Mordecai et al. found that COC users performed better during the active pill phase compared to the inactive pill phase (Mordecai et al. 2008). On the contrary, Mordecai et al. showed a trend toward a negative association between natural progesterone and verbal memory in non-users (Mordecai et al. 2008), and a recent systematic review found progesterone to be both beneficial and disruptive in relation to memory (Barros et al. 2015; Mordecai et al. 2008).

Mood deterioration is commonly reported as the reason for early discontinuation of COCs (Martell et al. 2023) and converging evidence from large population studies have linked COC use to increased risk of depression and suicide (Anderl et al. 2020; Edwards et al. 2022; Johansson et al. 2023; Skovlund et al. 2016, 2018). Notably, cognitive symptoms including decreased verbal memory capacity are common in patients with depression (Dam et al. 2021; Otte et al. 2016). Therefore, we wanted to investigate if subclinical mood symptoms could affect the association between COC use and verbal memory performance. We found no evidence that the participants’ self-reported level of mental distress differed between COC users and non-users, nor that it had an influence on their verbal memory performance. However, the participant’s TMD scores were very low, and we therefore cannot exclude that an association could be present in a clinical population. We also did not find any evidence for an effect of COC use on verbal affective memory bias, but evidence indicating that wordlist tasks are sensitive to measure affective memory bias is sparse (Dam et al. 2021). Thus, it is possible that VAMT-24/VAMT-26 is not sufficiently sensitive to detect subtle group differences in affective bias in verbal memory recall in healthy women.

Some studies suggest that the adolescent brain is more susceptible to hormonal influences (Brønnick et al. 2020; Cahill 2018; Skovlund et al. 2016, 2018). Suppression of endogenous sex hormones may affect hormone-dependent maturation of the adolescent brain, and consequently cognitive functioning and behaviour including verbal memory. However here, within a young adult population, we found no evidence of an age-dependent effect of COC use on verbal memory.

Understanding the neurobiological mechanisms by which hormonal contraception may affect cognition and mental health in some women is pivotal for protecting reproductive mental health. We recommend conducting large-scale randomized control studies on cognition and related brain biology in adolescents which consider the effect of different hormonal contraceptive types and which examine the effect of pill and menstrual cycle phases.

### Methodological considerations and limitations

To our knowledge, this is the largest study investigating the effect of COC use on verbal memory function in healthy women to date, and one of the first studies to examine bioavailable plasma estradiol levels in a large sample of naturally cycling women in the follicular phase.

Some limitations should be considered, when interpreting our results. Firstly, since a large proportion of women discontinue COC due to mood-related side effects, by studying prevalent users and not incident users, the population is selected on whether the women tolerate COC or not, which is likely to introduce a healthy user bias (Johansson et al. 2023). This may explain the tendency to the better performance in the COC users on short- and long-term memory retrieval, and it is likely to be the source of the conflicting result in the literature, why there is a need for randomized studies. Secondly, this is an observational study potentially biased by confounding. Thirdly, information on menstrual cycle phase on all naturally cycling women in the non-user group were not available, thereby limiting our ability to control for menstrual cycle phases in all analyses. However, most non-users with information provided on menstrual cycling phase were recruited during their follicular phase, and because of this, most non-users have been neuropsychologically tested during low levels of natural estrogen and progesterone. Fourthly, we chose a pragmatic pseudo-median split approach to investigate a possible age-dependent effect of COC use on verbal memory; however, our sample did not include women younger than 18 years of age and such potential effect may be more evident in another study population, e.g. adolescent women. Lastly, as this cohort consist of highly educated women, these findings may not be generalized to the broader population.

## Conclusions

We did not observe any difference in verbal memory in COC users relative to non-users. However, we did observe a non-significant trend in short- and long-term memory retrieval suggesting that COC use may induce slight pro-memory effects in healthy women, although such small improvements are likely to have little to no clinical relevance and potentially subject to a healthy user bias. Perhaps more importantly, we found no indication that COC use impairs verbal memory, which emphasizes that women who tolerate COCs well should not be concerned about potential adverse effects on memory linked to COC use.

## Supplementary Information

Below is the link to the electronic supplementary material. 

ESM1 (DOCX 880 KB)
