# Supplementary material for: Effect of combined oral contraceptive use on verbal memory function in healthy women
Source: Arch Womens Ment Health. 2025 May 19;28(5):1211–9. doi: 10.1007/s00737-025-01592-z (PMC12436564; doi:10.1007/s00737-025-01592-z)
Supplement: Supplementary file 1 — (DOCX 880 KB) [file 737_2025_1592_MOESM1_ESM.docx]

# Supplementary figures

**
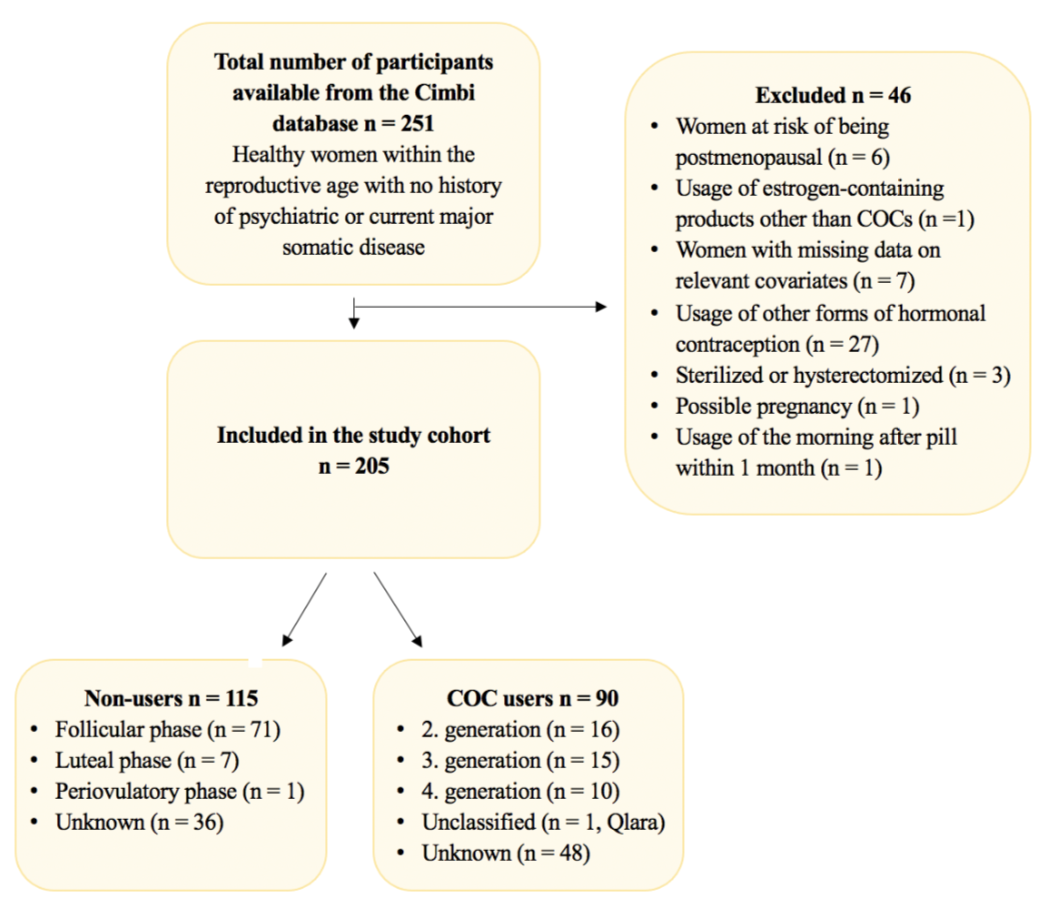
**

***Figure S1****: Overview of the data cleaning process including categorization of non-users into menstrual cycling phase based on menstrual cycling information obtained up 30 days from the cognitive testing and the count-back method, and COC users in generations according to the progestin of the combined oral contraceptive (COC). Data was extracted 4^th^ of January 2024. Center for Integrated Molecular Brain Imaging (Cimbi) database.*

**Figure S2: Difference in immediate recall (IMR) in COC users relative to non-users**

**
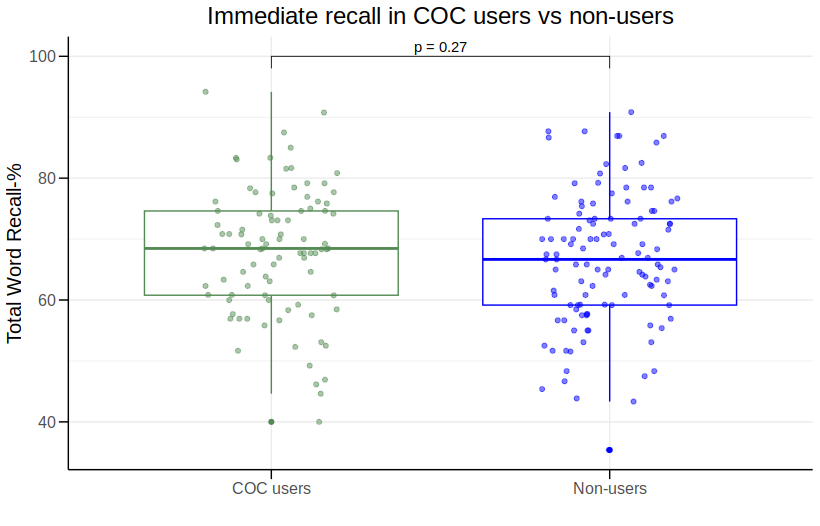
**

**Figure S2**: Total Word Recall-% in IMR 1-5 is calculated as percentage correctly recalled words across all valence categories (positive, negative, and neutral) across immediate recall conditions in COC users vs. non-users.

**Figure S3: Difference in short-term memory (STM) in COC users relative to non-users**


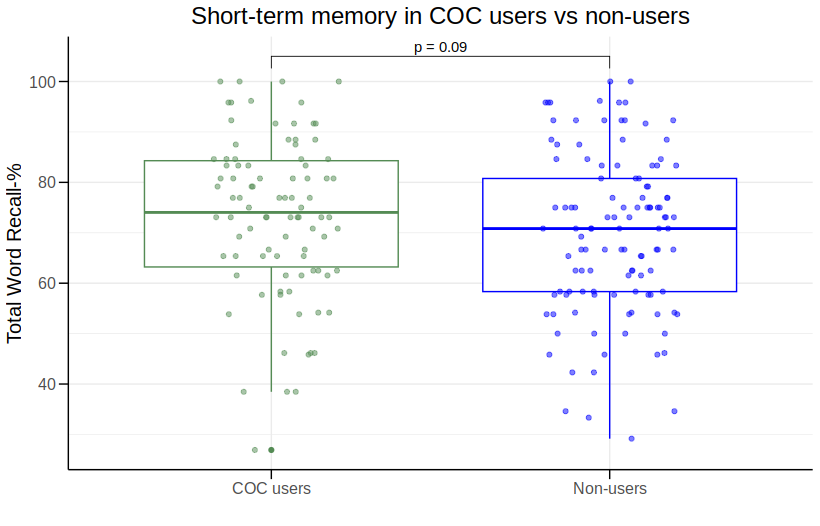


**Figure S3**: Total Word Recall-% in STM is calculated as percentage correctly recalled words across all valence categories (positive, negative, and neutral) in the short-term memory trial in COC users vs. non-users.

**Figure S4: Difference in long-term memory (LTM) in COC users relative to non-user**


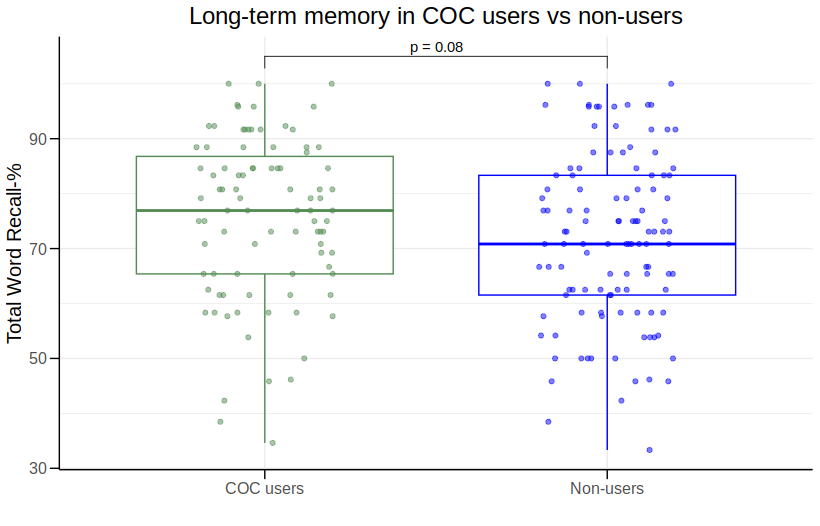


**Figure S4**: Total Word Recall-% in LTM is calculated as percentage correctly recalled words across all valence categories (positive, negative, and neutral) in the long-term memory trial in COC users vs. non-users.

**Figure S5: Verbal memory in hormonal IUD-users, COC users and non-users**


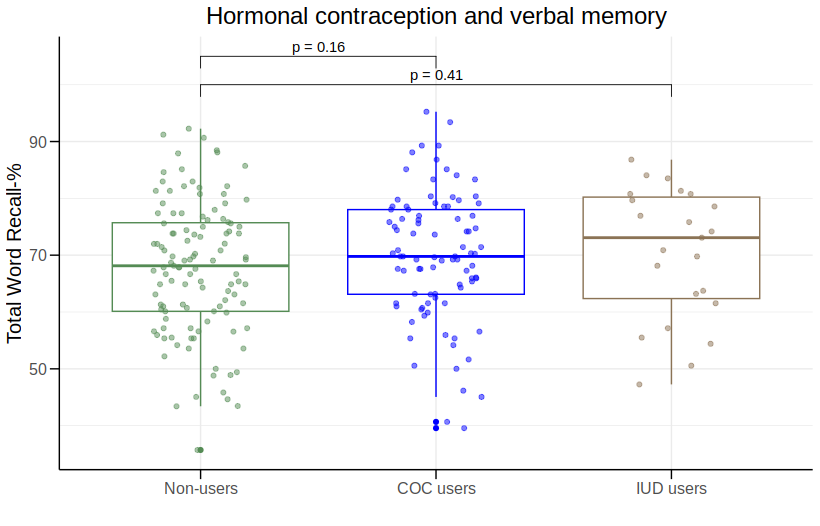


**Figure S5**: Total Word Recall-% in naturally cycling (NC) women (non-users), combined oral contraceptive (COC) users and hormonal intrauterine device (IUD) users.

**Figure S6: Difference in Total Word Recall-% in COC users relative to women in the follicular phase**


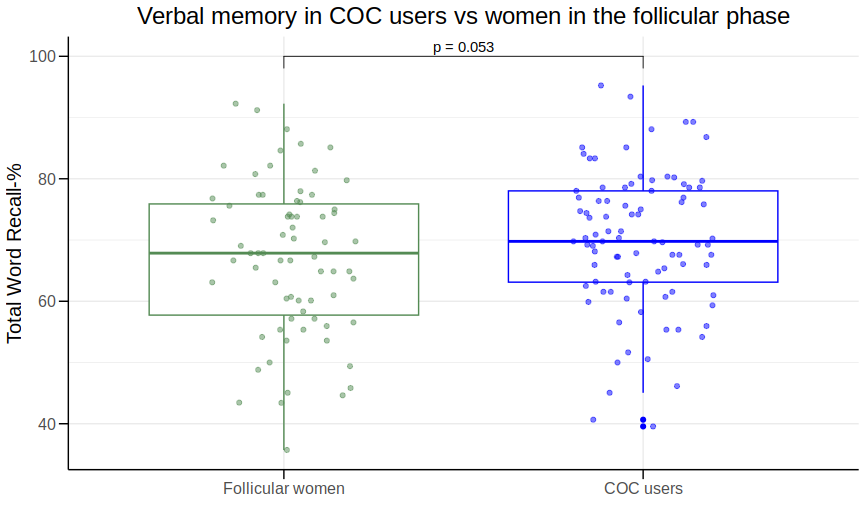


**Figure S6**: Total Word Recall-% is calculated as percentage correctly recalled words across all valence categories (positive, negative, and neutral) across immediate recall, short-term recall, and long-term recall conditions in COC users vs. women in the follicular phase.

| **Supplementary Table S1** |  |
| --- | --- |
|  |  |
| All study protocols |  |
| (KF)01-2006-20, (KF)23830, (KF)01280377, H-1-2010-085, H-15004506, H-15017713, H-16026898,  H-18038325, H-2-2010-108, H-2-2014-070, H-3-2012- 083, H-4-2011-103, H-4-2012-105. |  |

| **Supplementary Table S2** | |  |  |
| --- | --- | --- | --- |
| Association between immediate recall trials, short-term memory trial and long-term memory trial and COC use | | | |
| **VAMT trial** | *Estimate | 95% CI | *p* values |
| All  Immediate recall (IMR)  Short-term memory (STM)  Long-term memory (LTM) | 2.30  1.70  3.87  3.74 | [-0.94; 5.54]  [-1.33; 4.73]  [-0.56; 8.30]  [-0.47; 7.94] | 0.16  0.27  0.09  0.08 |
| **Estimate represents group differences in Total Word Recall-% in percentage across all trials, immediate recall (IMR) trials, short-term memory (STM) trial and long-term memory (LTM) trial in COC users relative to non-users* | | | |
| Association between verbal memory across all trials and COC use | | | |
| **Sensitivity analyses** | **Estimate | 95% CI | *p* values |
| Inclusion of TMD score in the regression model  Exclusion of those with unknown hormonal contraceptive status other than COCs | 2.14  2.33 | [-1.11; 5.40]  [-1.29; 5.94] | 0.20  0.21 |
|  |  |  |  |
| ***Estimate represents group differences in Total Word Recall-% in percentage across all trials in COC users relative to non-users* | | | |
|  |  |  |  |

| **Supplementary Table S3** | | |  |  |  | |  |  |
| --- | --- | --- | --- | --- | --- | --- | --- | --- |
| The means for correctly recalled negative and positive words in VAMT trials, expressed as percentages,  in each group | | | | | | | | |
| **VAMT trial** | Immediate recall (IMR) | | Short-term memory (STM) | | Long-term memory (LTM) | | | |
|  | Negative | Positive | Negative | Positive | Negative | Positive | | |
| Group |  |  |  |  |  |  | | |
| COC users | 65.2% | 66.8% | 69.9% | 70.4% | 70.9% | 76.1% | | |
| Non-users | 63.3% | 67.0% | 64.8% | 68.9% | 65.8% | 71.9% | | |
